# Influence of Urban Informal Settlements on Trace Element Accumulation in Road Dust and Their Possible Health Implications in Ekurhuleni Metropolitan Municipality, South Africa

**DOI:** 10.3390/toxics10050253

**Published:** 2022-05-17

**Authors:** Innocent Mugudamani, Saheed A. Oke, Thandi Patricia Gumede

**Affiliations:** 1 Department of Life Sciences, Central University of Technology, Bloemfontein 9301, Free State, South Africa; imugudamani@gmail.com (I.M.); TGumede@cut.ac.za (T.P.G.); 2 Department of Civil Engineering, Centre for Sustainable Smart Cities, Central University of Technology, Bloemfontein 9301, Free State, South Africa

**Keywords:** informal settlement, trace elements, road dust, health implications, carcinogenic, non-carcinogenic

## Abstract

The study was aimed at assessing the influence of urban informal settlement on trace element accumulation in road dust from the Ekurhuleni Metropolitan Municipality, South Africa, and their possible health implications. The concentration of major and trace elements was determined using the wavelength dispersive XRF method. The major elements in descending order were SiO_2_ (72.76%), Al_2_O_3_ (6.90%), Fe_2_O_3_ (3.88%), CaO (2.71%), K_2_O (1.56%), Na_2_O (0.99%), MgO (0.94%), MnO (0.57%), TiO_2_ (0.40%), and P_2_O_5_ (0.16%), with SiO_2_ and P_2_O_5_ at above-average shale values. The average mean concentrations of 17 trace elements in decreasing order were Cr (637.4), Ba (625.6), Zn (231.8), Zr (190.2), Sr (120.2), V (69), Rb (66), Cu (61), Ni (49), Pb (30.8), Co (17.4), Y (14.4), Nb (8.6), As (7.2), Sc (5.8), Th (4.58), and U (2.9) mg/kg. Trace elements such as Cr, Cu, Zn, Zr, Ba, and Pb surpassed their average shale values, and only Cr surpassed the South African soil screening values. The assessment of pollution through the geo-accumulation index (Igeo) revealed that road dust was moderately to heavily contaminated by Cr, whereas all other trace elements were categorized as being uncontaminated to moderately contaminated. The contamination factor (CF) exhibited road dust to be very highly contaminated by Cr, moderately contaminated by Zn, Pb, Cu, Zr, and Ba, and lowly contaminated by Co, U, Nb, Ni, As, Y, V, Rb, Sc, Sr, and Th. The pollution load index (PLI) also affirmed that the road dust in this study was very highly polluted by trace elements. Moreover, the results of the enrichment factor (EF) categorized Cr as having a significant degree of enrichment. Zn was elucidated as being minimally enriched, whereas all other trace elements were of natural origin. The results of the non-carcinogenic risk assessment revealed a possibility of non-carcinogenic risks to both children and adults. For the carcinogenic risk, the total CR values in children and adults were above the acceptable limit, signifying a likelihood of carcinogenic risk to the local inhabitants. From the findings of this study, it can be concluded that the levels of trace elements in the road dust of this informal settlement had the possibility to contribute to both non-carcinogenic and carcinogenic risks, and that children were at a higher risk than the adult population.

## 1. Introduction

The urban landscape of most developing nations has experienced a proliferation of informal settlements over many years [1], and the majority of Sub-Saharan African urban inhabitants (~55%) now reside in informal settlements [2]. Informal settlements are regarded as being neglected portions of cities where housing and living conditions are terribly poor. They vary from overcrowded, contaminated dwellings to inadvertent squatter locations with no legitimate rights, distributed at the edge of cities [3]. They are seriously characterized by man-made activities such as household heating, the combustion of coal and oil, industrial processes, unplanned construction, demolition activities, road weathering, poor waste management, the burning of waste, and dense traffic [4,5]. Such anthropogenic activities lead to the accumulation of trace elements on buildings, plants, in air, soil, water, and on road dust. Trace elements are not decomposable, and consequently, they persist for long periods of time in the environment [6]. Road dust refers to the re-suspended particulate matter found on roads, mostly in troughs. It sometimes comprises of soil and sand particles that are assorted with litter, and rubble that becomes airborne due to traffic movements [7]. As a results of traffic movements, road dust is frequently raised up, settled, and raised again, to a certain elevation, which exposes residents to any trace elements that are available in such dust [8]. Some of the trace elements connected to road dust are As (arsenic), Ba (barium), Co (cobalt), Cr (chromium), Cu (copper), Ni (nickel), Pb (lead), Zn (zinc), Zr (zirconium), [9] Nb (niobium), Rb (rubidium), Sc (scandium), Sr (strontium), Th (thorium), V (vanadium), and Y (yttrium). Exposure to road dust containing these trace elements, either through inhalation, ingestion, or dermal contact absorption [10] may ultimately lead to serious health implications on the local residents. These include medical conditions such as cancer, miscarriages, hearing and visual impairment, asthma, renal failure, high blood pressure, headaches and dizziness, problems of reproductive systems, cardiovascular disorder, writhing, ataxia, skin and eye irritation, and lung granulomas [11,12]. In South Africa, informal settlements are home-based areas to masses of households, and Ekurhuleni is a metropolitan municipality with roughly 26 percent of its inhabitants residing in informal settlements [13]. These areas are characterized by a lack of proper sanitation, dense traffic, human activities such as a high population, unplanned construction, poor waste management, unregulated waste incineration, charcoal burning, and sewage runoff onto the streets. All of these man-made activities may influence the proliferation of trace elements in road dust, and thus endanger the health of the local inhabitants. Therefore, studying the influence of urban informal settlements on trace element accumulation in road dust is not only an essential aspect of assessing the quality of urban informal settlement settings, but also of protecting the health of the local residents. Despite the available knowledge on the possible effects of trace elements in road dust on public health in South Africa and in other parts of the world, overpopulated informal settlements in South Africa are lacking scientific data on the concentration of trace elements and their associated health risks. Therefore, this study aim to fill in this lacuna by determining the concentration of trace elements, assessing their pollution levels and possible sources, evaluating the health risks, and determining possible health implications that are associated with trace element exposure in road dust. The findings of this study will provide scientific knowledge on the levels of trace elements in road dust, and create awareness about the potential health risks associated with trace element exposure for populations living in poor urban informal settlements.

## 2. Materials and Methods

### 2.1. Study Area

**Figure 1:**
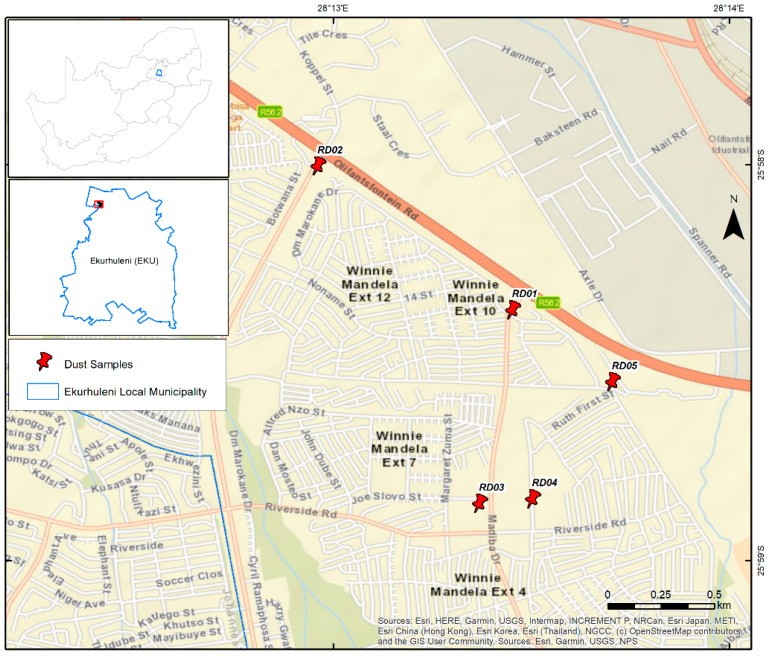
Study area and the location of the sampling site.

**Figure 2:**
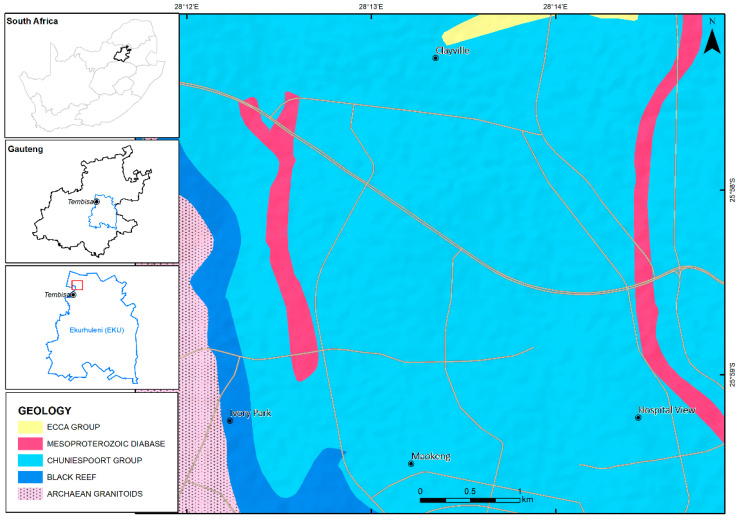
Geological map of the study area.

The study area is situated within Ekurhuleni Metropolitan Municipality in Gauteng Province, South Africa (Figure 1). The area is positioned 15 km north of Kempton Park City Centre, and approximately 39.4 km south of Pretoria. It consists of 41,581 households with a population of 91,646, and is mostly dominated by approximately 99% Black Africans over a surface area of 5.43 km^2^. The area experiences some rainfall, mainly during summer. It is frequently characterized by an average mean annual rainfall of 60 mm. January is considered to be the wettest month, with an average of approximately 125 mm. The month of July is the driest month, with an average of approximately 4 mm. Furthermore, the average mean annual temperature is 16 °C, with January being the warmest, at an average of 20.1 °C, whereas June is considered to be the coldest month, at an average of 10.1 °C. The study is characterized by human activities such as a high population, unplanned construction, poor waste management, unregulated waste incineration, charcoal combustion, firewood, and sewage waste runoff onto the streets. The settlement is decorated by barbecuing markets along the street, which bisect the settlement. Furthermore, the community transportation system functions with taxicabs, which are the most commonly used method of conveyance by inhabitants. Traffic congestion is noteworthy during the early morning and late afternoon peak hours on the main road that bisect the settlement. There is also an industrial area that is situated approximate 2 to 3 km away from the settlement, which acts as a source of employment for the residents. The area is underlain by Archean Cratonic rocks allocated to the Johannesburg Dome, also recognized as the halfway house or the Johannesburg-Pretoria Dome. The Johannesburg Dome is a dome-like window of ancient granitoid (approximately 750 km^2^) positioned in the middle part of the Kaapval Craton. It consist of black reef formation which form the base of the Transvaal Supergroup, which is an outcropping to the north-eastern, northern, and north-western margin of the inlier, and un-conformably overlies the granitoids and greenstones. It also comprises of trondhjemitic and tonalitic granitic rocks intruded into mafic-ultramafic greenstones. Furthermore, it encompasses some hornblende-amphibolites dykes and dolomites of the Chuniespoort group, as shown in Figure 2 [14].

### 2.2. Sample Collection and Chemical Analysis

The study area is characterized by different functional areas that include commercial, residential, roadway, taxi rank, and leisure parks/playing grounds. Five (5) road dust samples were collected from these different functional areas, using a random sampling method. The samples were exactly collected at one of the major roads that bisect the settlement, near the park, at the taxi rank, next to the primary school, and at the shopping mall, in order to cover all of the functional areas around the settlement. The points were recorded using GPS, as detailed in Table 1. A sampling campaign was conducted in June 2021, which is a dry month. The road dust particles within a 5 m range of the chosen sampling point were collected by sweeping with a brush and dustpan. A randomly selected sample was collected on a paved surface, and the sampling points are presented in Figure 1. Unrelated material such as litter and debris were taken out from the samples in the course of sampling. To avoid cross-contamination, equipment was cleaned after every location. The samples were then transferred into the plastic sample bags, labelled, and conveyed to the laboratory.

A total of 10 g of sample was ground to a particle size of less than 200 mesh. The 10 g sample was then heated to 110 °C to dehydrate and devolatilize the sample, and then to 1050 °C, which breaks down minerals such as carbonates. This was conducted to determine the total loss on ignition, or the total gain on ignition. Then, a flux of 0.2445 g of La_2_O_3_, 0.705 g of Li_2_B_4_O_7_, 0.5505 g of Li_2_CO_3_, and 0.02 g of NaNO_3_ was added to 0.28 g of sample. The mixture was then heated to 1000 °C for approximately 5 min until a consistent fluid was formed within a Pt crucible. The fluid was then poured into a mold and pressed to form the disc. The application used to measure the majors was “IGS majors”. It was created using the following standards: DR-N, JB-2, JF-2, JG-2, JGB-1, K8000, MA-N, MICA-FE, MRG-1, NIM-S, SARM4, SARM5, SARM6, SARM43, SARM44, SARM47, SARM48, SARM50, SARM52, SY-2, and UB-N for quality control.

To analyze the trace elements, 8 g of sample was added to 3 g of Hoechst wax (C_6_H_8_O_3_N_2_). It was then mixed for 20 min in a Turbula mixer to ensure that the sample was mixed until it was homogeneous. The mixture was then pressed to pressures of greater than 395 N/m. The calibrated application used for the trace elements analysis was ‘UIC traces’, and for the analysis of Na, it was ‘Sodium only’. The standards used to calibrate the ‘UIC traces’ included: ASK-2, ASK-3, BE-N, BHVO-1, BR, GA, GH, JA-1, JB-1, JB-2, JDO-1, JG-1, JG-2, JLS-1, JP-1, JR-1, JR-2, K8000, MA-N, MICA-FE, MICA-MG, MRG-1, NIM-D, NIM-G, NIM-L, NIM-N, NIM-P, NIM-S, RGM-1, SY-2, TRABS-001, TRABS-002, TRABS-003, TRABS-004, TRACE-000, TRACE-001, TRACE-002, TRACE-003, TRACE-004, TRACE-005, TRACE-006, TRACE-007, TRACE-008, TRACE-009, TRACE-010, TRACE-011, TRACE-012, TRACE-013, TRACE-014, TRACE-015, TRACE-016, TRMAC-001, TRMAC-002, TRMAC-003, TRMAC-004, TRMAC-005, TRMAC-006, UB-N, and VS-N for the quality control. CaO, TiO_2_, and Fe_2_O_3_ were measured to correct for line overlaps. The standards used to calibrate ‘Sodium only’ included: AN-G, BR, FK-N, G2, GA, GH, GS-N, GSP-1, JG-1, NIM-G, NIM-N, NIM-P, NIM-S, SARM39, and SY-2. Blank samples and duplicates were also used to determine precision and bias. The level of inconsistency was determined to be <10%. The WD-XRF machine used in this study was a Rigaku-Primus IV, with an Rh tube. The software used for the machine was ZXS, and the results are quantitative results.

### 2.3. Pollution Assessment of Trace Elements in Road Dust

#### 2.3.1. Geo-Accumulation Index (Igeo)

This involves matching the level of the determined trace element to the average shale value or background levels [15,16]. This was computed using Equation (1):

Igeo = log_2_ (Cn/1.5 Bn) (1)

where Cn signifies the measured concentration in this study, and Bn represents the geochemical background value or an average shale value of an element of interest. A constant of 1.5 is presented to reduce the effect of possible variations in the background values that may be attributed to lithological differences in the sediments or soil [17]. Igeo values divide soil into different quality classes: Class 0 (Igeo ≤ 0) uncontaminated; Class 1 (0 < Igeo ≤ 1) uncontaminated to moderately contaminated; Class 2 (1 < Igeo ≤ 2) moderately contaminated; Class 3 (2 < Igeo ≤ 3) moderately to heavily contaminated; Class 4 (3 < Igeo ≤ 4) heavily contaminated; Class 5 (4 < Igeo ≤ 5) heavily to extremely contaminated; and Class 6 (Igeo > 5) extremely contaminated [15].

#### 2.3.2. Contamination Factor (CF) and Pollution Load Index (PLI)

The contamination factor permits the evaluation of soil pollution by taking into consideration the content of trace elements in the soil and its background values or average shale value [15]. This was calculated using Equation (2):

CF = C sample/C background(2)

where C sample represents the concentration of an element of interest and C background is the metal background concentration or average the shale value of an element of interest. Consistent with Addo et al. [18], the CF values were categorized into four clusters: CF < 1 (low contamination), 1 ≤ CF < 3 (moderate contamination), 3 ≤ CF ≤ 6 (considerable contamination), and CF > 6 (very high contamination). The PLI was then calculated using the values of CF. This verifies how environmental conditions have deteriorated due to a rise in metal concentration [19], using Equation (3):

PLI = ^n^ √ (CF1 × CF2 × CF3 × … CFn) (3)

where n represents the number of trace elements detected in this study, and CF connotes the contamination factor computed using Equation (2). According to Kowalska et al. [15], PLI classifies site quality as: 0 < PLI ≤ 1 (unpolluted), 1 < PLI ≤ 2 (moderately to unpolluted), 2 < PLI ≤ 3 (moderately polluted), 3 < PLI ≤ 4 (moderately to highly polluted), 4 < PLI ≤ 5 (highly polluted), and 5 < PLI (very highly polluted).

#### 2.3.3. Enrichment Factors (EF)

The EF was used to differentiate between elements originating from human activities and natural sources [20]. This was computed using Equation (4) by comparing the concentration of an element in a sample with its concentration in the average shale value. Scandium (Sc) was used as the reference element [21].

EF = (E/R) sample/(E/R) background(4)

where E is the concentration of an element of interest, R is a reference element of crustal material (Sc), and (E/R) sample is the concentration ratio of E to R in the collected samples, and (E/R) background is the concentration ratio of E to R in the Earth’s crust. The EF is categorized into five classes: EF < 2 (depletion to minimal enrichment), EF = 2–5 (moderate enrichment), EF = 5–20 (significant enrichment), EF = 20–40 (very high enrichment), and EF > 40 (extremely high enrichment) [20,21].

### 2.4. Human Health Risk Assessment of Trace Elements in Road Dust

#### 2.4.1. Non-Cancer Risk Assessment

The average daily dose (ADD) of each analyzed trace element through ingestion, inhalation, and dermal contact was calculated using Equations (5)–(7) [22,23].

ADD_ing_ = C × IngR × CF × EF × ED/BW × AT (5)

ADD_inh_ = C × InhR × EF × ED/BW × AT × PEF (6)

ADD_derm_ = C × SA × CF × SL × ABS × EF × ED/BW × AT (7)

where ADD_ing_ signifies the average daily ingestion (mg/kg/day) amount for an element, ADD_inh_ indicates the average daily inhalation (mg/kg/day) amount for an element, and ADD_derm_ specifies the average daily dermal (mg/kg/day) exposure amount of metal, and their values are presented in Table 2. Non-cancer risk was then evaluated from the hazard quotient (HQ) for each trace element by dividing the ADD calculated in Equations (5)–(7) with a particular reference dose (Rfd), using Equation (8):

HQ = ADD/RfD(8)

HQ > 1 suggests a possibility of health effects, while HQ < 1 shows no possibility of health effects [24]. Furthermore, the hazard index (HI) was then calculated by adding the HQ of the three exposure pathways for a corresponding element [25], using Equation (9):

HI = (HQ) ing + (HQ) inh + (HQ) derm(9)

A HI value < 1 describes a very low risk, a HI value between 1 and 4 shows that the risk effects are possible, and HI values > 4 describe a high risk [25].

**Table 2:** Exposure factors for dose models.

Items	Parameter	Meaning	Unit	Value		References
				Children	Adult	
Basic parameter	C	Concentration of a metal	mg/kg	This study	This study	[9,23,26]
	D	Daily dose	mg/kg			[9,23,26]
	CF	Conversion factor	kg/mg	1 × 10^−6^	1 × 10^−6^	[9,23,26]
Exposure behavioral parameter	ED	Exposure duration	years	6	24	[9,23,26]
	BW	Body weight	kg	15	55.9	[9,23,26]
	EF	Exposure frequency	days/year	350	350	[9,23,26]
	AT	Average time (carcinogen)	days	365 × 70	365 × 70	[9,23,26]
		Average time (non-carcinogen)	days	365 × ED	365 × ED	[9,23,26]
Digestive tract/inhalation	InhR	Inhalation rate	m^3^/kg	5	20	[9,23,26]
	IngR	Ingestion rate	mg/kg	200	100	[9,23,26]
	PEF	Particle emission factor	m^3^/kg	1.32 × 10^9^	1.32 × 10^9^	[9,23,26]
Skin contact	SL	Skin adherence factor	mg/cm^2^	1	1	[9,23,26]
	SA	Skin surface area	cm^2^	1800	5000	[9,23,26]
	ABS	Dermal absorption	-	0.001	0.001	[9,23,26]

#### 2.4.2. Cancer Risk Assessment

The lifetime average daily dose (LADD) of each of the analyzed elements was also calculated for ingestion, inhalation, and dermal exposure pathways, using Equations (10)–(12) [9].

LADD_ing_ = C × CF × EF/AT × (IngR_child_ × ED_child_/BW_child_ + IngR_adult_ × ED_adult_/BW_adult_) (10)

LADD_inh_ = C × EF/AT × PEF × (InhR_child_ × ED_child_/BW_child_ + InhR_adult_ × ED_adult_/BW_adult_) (11)

LADD_derm_ = C × CF × EF × SL × ABS/AT × (SA_child_ × ED_child_/BW_child_ + SA_adult_ × ED_adult_/BW_adult_) (12)

where, LADD_ing_ connotes the lifetime average daily ingestion (mg/kg/day) amount of a metal, LADD_inh_ implies the lifetime average daily inhalation (mg/kg/day) amount of an element, and LADD_derm_ indicates the lifetime average daily dermal (mg/kg/day) exposure amount of a metal, and their values are presented in Table 2 [9,23,26,27,28,29]. After calculating the LADD of each exposure pathway, a lifetime cancer risk (CR) was then computed by multiplying the LADD with an equivalent slope factor (SF) using Equation (13). The permissible risk usually ranged from 10^−6^ to 10^−4^: very low (<10^−6^), low (10^−6^–10^−5^), medium (10^−5^–10^−4^), high (10^−4^–10^−3^), and very high (>10^−3^) [30].

CR = LADD × SF(13)

### 2.5. Statistical Methods

The laboratory results were analyzed using Statistical Package for Social Sciences (SPSS) from Microsoft Excel. The data were presented as the minimum, maximum, average mean, and standard deviation. Furthermore, to determine the possible relationship between the elemental concentration and the possible source of origin, Pearson’s correlation coefficient and a one way analysis of variance (ANOVA) were adopted.

## 3. Results and Discussion

### 3.1. Concentration of Major Elements in Road Dust

The descriptive statistics of major elements is summarized in Table 3, with the average shale values (ASV). The average mean concentrations of SiO_2_ and P_2_O_5_ were higher than their average shale values. A higher silica content may expose the community to serious health implications, such as damaged lung tissue [20]. Its higher concentration in road dust may be associated with its hardness, which makes it difficult to undergo physical weathering [31]. Additionally, the dominance of quartz in road dust may also be linked to the geographical location of the study area, which falls under the Johannesburg dome, which is underlain with sedimentary rocks of the Witwatersrand and Venterdorp Supergroup.

The concentration of P_2_O_5_, which was also higher than its average shale value, might have been influenced by poor waste management practices in this informal settlement, particularly waste containing phosphate [31]. Runoff from gardens, illegal dumping, and nearby roadside soil polluted by phosphate used in different agricultural activities might have exacerbated the concentration of P_2_O_5_ in road sediments. Exposure to dust particles containing high concentrations of P_2_O_5_ may lead to the risk of respiratory distress, and problems of the liver, kidneys, and brain [32].

Furthermore, the average mean concentration of major elements such as Al_2_O_3_, K_2_O, MgO, MnO, and CaO were below their average shale values, suggesting that they are of natural origin and were comparable to the findings of Li et al. [33] in Xi’an city, China. Although these major elements were below their average shale values, their concentrations may possible rise in the near future, due to daily increases in the population and uncontrolled waste generation in this poor informal settlement.

**Table 3:** Composition of major elements in road dust samples (Wt. %).

Elements	RD01	RD02	RD03	RD04	RD05	Min-Max	Mean	±SD	ASV
Al_2_O_3_	7.22	8.43	6.21	5.9	6.72	5.9–8.43	6.9	0.99	15.4
CaO	4.23	3.62	1.97	1.09	2.66	1.09–4.23	2.71	1.26	3.1
Fe_2_O_3_	3.41	3.52	3.68	4.11	4.67	3.41–4.67	3.88	0.52	4.02
K_2_O	1.43	2.44	1.27	1.45	1.22	1.22–2.44	1.56	0.5	3.24
MgO	1.18	1.15	0.72	0.32	1.33	0.32–1.33	0.94	0.41	2.44
MnO	0.42	0.24	0.66	1.03	0.51	0.24–1.03	0.57	0.3	trace
Na_2_O	0.98	1.89	0.71	0.76	0.62	0.62–1.89	0.99	0.52	1.3
P_2_O_5_	0.12	0.37	0.11	0.08	0.12	0.08–0.37	0.16	0.12	0.14
SiO_2_	69.17	65.37	75.07	79.92	74.25	65.37–79.92	72.76	5.62	58.11
TiO_2_	0.36	0.35	0.46	0.35	0.48	0.35–0.48	0.4	0.06	0.65
LOI	11.3	10.52	7.47	4.98	6.61	4.98–11.3	8.18	2.67	-

Notation: LOI = loss on ignition; SD = Standard deviation; ASV = Average shale value; Min = Minimum; Max = Maximum; Average shale value [34].

### 3.2. Concentration of Trace Elements in Road Dust

The concentration of trace elements presented in Table 4 were ranging as Cr > Ba > Zn > Zr > Sr > V > Rb > Cu > Ni > Pb > Co > Y > Nb > As > Sc > Th > U. Cr, Cu, Zn, Zr, Ba, and Pb were above their average shale values [35], which support the findings of Cai and Li [8] in the street dust of Shijiazhuang, China. When compared with the South African soil screening values [36] for metals in informal settlements only Cr was above this value. This outcome corresponds to the findings of the study conducted by Kamunda et al. [37] in soils from the Witwatersrand Gold Mining Basin, South Africa. The high levels of these trace elements might have been influenced by man-made activities. Dense traffic, which is mostly seen during the early hours and late hours of the day, mostly in tar roads that bisect the settlement, is one of the factors that influence the accumulation of trace elements. Vehicle exhaust is associated with Zr [38], while tire rubber, break wear re-suspended particles, and fuel combustion are sources of Zn [31]. The accumulation of Cu in street dust is associated with brake pad wear [31], while Pb mostly emanated from brake friction, batteries, and gasoline [39].

According to Moryani et al. [40], Cr may be released from the combustion of lubricants and fuel. Charcoals is used most of the time in this poor informal settlement for household warming and cooking, and street barbecuing may also distribute Cr around the settlement through fly ash. According to Cui et al. [41], volatile condensing elements such as Cr are enriched in fine fly ash. Unplanned construction in this informal settlement, which occurs most of the time, may also release dust containing Co, possibly from materials containing cobalt, such as alloys and paints [42]. Furthermore, the dumping of waste in any available space or adjacent to the streets may release elements such as Ba. Waste materials such as ceramics, glass, or plastics are considered to be possible sources of Ba [43]. Tires and brakes are also sources of Ba.

When trace elements in road dust were compared with other cities around the world, as shown in Table 5, there were variations in their concentrations. This variation may be attributed to various factors such as a high population, unregulated waste burning, unplanned construction, charcoal burning and the use of firewood, emissions from nearby industrial areas, sewage waste, and poor waste management practices in the settlement. Salah et al. [44] agrees that the accumulation of trace elements in different regions may be influenced by factors such as the type of man-made activities. According to Shi et al. [42], the population level and stages of development may also influence differences in trace element concentration.

### 3.3. Pollution Assessment and Identification of Sources of Trace Elements in Road Dust

#### 3.3.1. Geo-Accumulation Index (Igeo)

The average Igeo value showed that the road dust in the informal settlement was moderately contaminated by Cr, which corresponds with the study by Shi et al. [42], who reported road dust in Xian, China, to be moderately contaminated by Cr. It was also uncontaminated to moderately contaminated by Co, Cu, Zr, V, As, Pb, Ni, Nb, Zn, Ba, Sc, U, Y, Th, Rb, and Sr, which is similar to the findings of other researchers. Shi et al. [42] observed road dust to be uncontaminated to moderately contaminated by Zn, and Cu in Xian, China. Another study in Maha Sarakham Municipality, Thailand, reported road dust to be uncontaminated to moderately contaminated by Cu [17]. In Tianshui, China, Tan et al. [50] witnessed road dust to be uncontaminated to moderately contaminated by Cu and As, while in Dhaka city, Bangladesh, Rahman et al. [45] discovered road dust to be uncontaminated to moderately contaminated by Rb.

**Figure 3:**
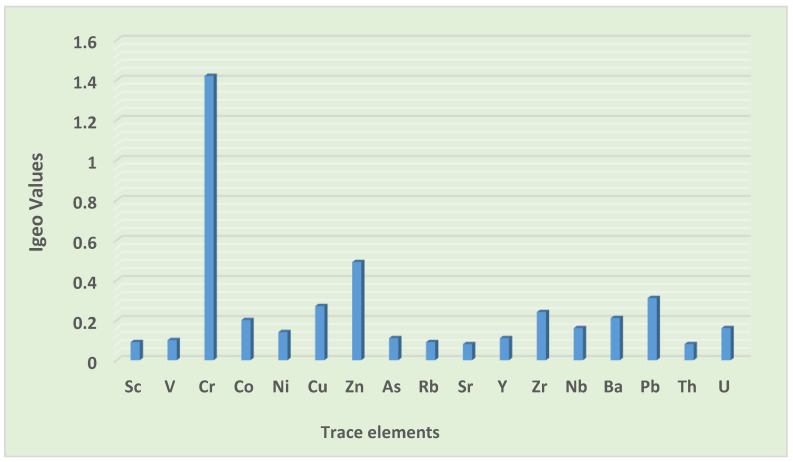
Trace element contamination levels in road dust based on Igeo values.

The inclusive Igeo values as presented in Table 6 were as follows: Cr > Zn > Pb > Cu > Zr > Ba > Co > U > Nb > Ni > Y > As > V > Sc > Rb > Sr > Th. Cr was the leading element, indicating possible anthropogenic sources (Figure 3). The possible anthropogenic sources of Cr in this study may be flying ashes from the combustion of charcoal used for indoor warming, cooking, and street barbecuing. Furthermore, the corrosion of vehicular parts, and the combustion of lubricants and fuel are also considered to be sources of Cr [40]. On the basis of the Igeo average values, Cr in this study is an element of concern, and its exposure at high concentrations may trigger serious health implications for the local inhabitants, particularly vulnerable groups such as children, elders, and pregnant women.

#### 3.3.2. Contamination Factor (CF) and Pollution Load Index (PLI)

The CF and PLI were adopted to determine the degree of pollution, and to verify how environmental conditions have deteriorated due to the rise in trace element concentrations. The CF average mean value for Cr was above 6, indicating a very high rate of contamination from human activities. This outcome surpasses the findings of Dat et al. [51] for street dust in a metropolitan area of Southern Vietnam, which recorded considerable contamination by Cr. Moderate contamination was noted for elements such as Zn, Pb, Cu, Zr, and Ba, signifying a natural origin with the moderate influence of anthropogenic activities. Similarly, Al-Dabbas et al. [52] also witnessed the moderate contamination of Pb and Zn in the street dust of Diwaniya, Iraq. The majority of trace elements such as Co, U, Nb, Ni, As, Y, V, Rb, Sc, Sr, and Th were classified as having low levels of contamination resembling a natural origin, and this agrees with a study conducted in Bolgatanga Municipality, Ghana [10], which observed a low level of contamination by Co, Ni, and As in road dust, and in the street dust of Diwaniya, Iraq, which was contaminated with a low level of V [52].

**Figure 4:**
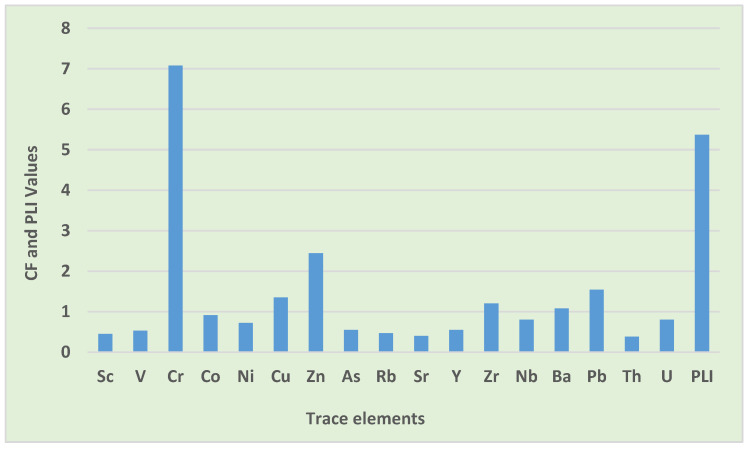
Contamination factor and pollution load index of individual trace elements in road dust.

As summarized in Table 7, the overall contamination factor values descended in the order of Cr > Zn > Pb > Cu > Zr > Ba > Co > U > Nb > Ni > As > Y > V > Rb > Sc > Sr > Th. As depicted in Figure 4, Cr was the leading pollution contributor amongst trace elements, possibly originating from traffic and charcoal burning, which is practiced daily for household purposes such as indoor warming and cooking. Furthermore, the use of charcoal by street vendors, and emissions from nearby industrial areas, may also be a possible source of Cr. The outcomes of the PLI results showed that road dust was very highly polluted, a similar outcome to the study conducted in the street dust of Ho Chi Minh City, Vietnam [51]. This outcome of PLI may have been influenced by factors such as daily heavy traffic within the study area [31,40,43]. Furthermore, runoff from sewage and uncollected waste materials that lie adjacent to the streets, unregulated waste incineration, and coal fly ashes might also be a contributor to the level of trace elements in road dust. The results of the contamination factor and pollution load index agree with the results of the Igeo accumulation index showing that Cr is an element of public concern in this study, and that remediation and regular monitoring are highly advocated.

#### 3.3.3. Enrichment Factor (EF)

To compute the EF values, the average shale values were used as the background concentration, and scandium (Sc) was chosen as a reference metal. The results of EF presented in Table 8 showed Cr to be of significant enrichment, possibly from human activities that matched the findings of the study conducted in the road dust of Katowice and Wroclaw, Poland [53], and they surpassed the findings observed by Cai and Li [8] in the street dust of Shijiazhuang, China, who reported Cr to be of minimal enrichment. Moderate enrichment was reported for Zn, indicating anthropogenic sources. These outcomes are better than the study conducted in Lagos metropolis, Nigeria [54], which observed a very high enrichment of Zn. Other trace elements, Pb, Cu, Zr, Ba, Sc, Co, U, Nb, Ni, Y, As, V, Rb, Sr, and Th were classified as having minimal enrichment, signifying a natural origin, in agreement with the study conducted on the road dust of Dhaka city, Bangladesh [55], which reported Pb, and As to be of minimal enrichment. In the road dust of Bolgatanga Municipality, Ghana, Cu, Zn, Pb, Ni, As, and Co were classified as having minimal enrichment [10]. The geology of the area, the development of the area, the selection of reference materials in calculating EF, and the selection of an element of reference may have an influence on the results of EF [56].

**Figure 5:**
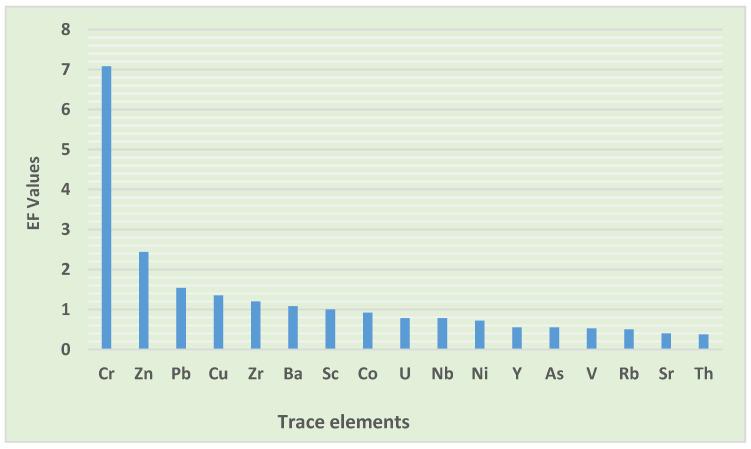
Enrichment factor values of trace elements in road dust.

The values of EF were as follows: Cr > Zn > Pb > Cu > Zr > Ba > Sc > Co > U > Nb > Ni > Y > As > V > Rb > Sr > Th. Cr was the major contributor to pollution, followed by Zn, which shows an influence from anthropogenic activities (Figure 5). Human activities such as waste incineration, heavy traffic, poor waste management, coal fly ashes, sewage waste run-off onto the streets, and emissions from nearby industrial areas might be possible anthropogenic sources in this informal settlement. Other researchers also agree that sewage and the incineration of plastics waste may release Zn into urban areas [57], whereas the corrosion of vehicular parts may be the source of Cr [10]. From the results of EF, it can be concluded that the high level of Cr in road dust needs serious attention. Other trace elements were of crustal origin. The natural sources of this trace element may be attributed to the geology of the area, precipitation, or wind-borne soil particles [58].

#### 3.3.4. Pearson Correlation Coefficient Analysis

Pearson’s correlation coefficient was performed to establish trace element relationships and to determine their common sources of origin. A correlation matrix of trace elements in road dust samples generated a diverse relationship between the elements. From the correlation analysis in Table 9, a sufficiently high degree of correlation, a moderate degree, and no positive correlation results were observed. A strong correlation was witnessed between pairs of Ni–Cr (0.97), Zn–Cu (r = 0.99), Co–V (r = 0.83), Co–Cr (r = 0.89), Ni–Co (r = 0.81), As–Sc (r = 0.78), Sr–Sc (r = 0.88), Sr–As (r = 0.88), Zr–V (r = 0.81), Pb–Cr (r = 0.89), Pb–Co (r = 0.87), and Pb–Ni (0.77). According to Weissmannova et al. [59], high levels of correlation among trace elements indicates the same sources of pollution, or anthropogenic sources. Therefore, the high degree of correlation between these trace elements in this study is suggestive of the same source of pollution, potentially from anthropogenic activities such as unplanned construction, coal fly ashes, poor waste management, the burning of waste, and dense traffic. Elements such as Zn, Ni, and Cr may be attributed to the burning of fuel [31,40]. Cu and Pb may be associated with brake pad wear and brake friction [31]. Coal burning releases As and Sr [43,45]. Trace elements such as Zr potentially emanate from vehicle exhaust [38], Co from construction materials such as paints [42], and V from oil combustion [43]. Furthermore, the contribution of the concentration of Sc in road dust is understood to be from soil re-suspension, as they are crustal elements [60].

#### 3.3.5. Statistical Analysis of Trace Elements

A one-way ANOVA was performed to test the difference between the concentrations of elements in road dust samples. The analysis of variance revealed the *p*-value to be 5.09 × 10^−20^, as shown in Table 10. The *p*-value was less than the alpha level of 0.05, demonstrating significant differences (*p* < 0.05). This outcome signifies that the trace element pollutants were not from common anthropogenic sources, which is comparable with the study conducted in Yola, Nigeria [61]. In this informal settlement vehicle emissions, re-suspended dust, construction dust, coal fly ashes, emissions from nearby industrial areas, sewage waste, waste burning, or poor waste management may be considered as being possible sources of these trace elements.

### 3.4. Non-Carcinogenic and Carcinogenic Health Risk Assessments of Trace Elements in Road Dust

The health risk assessment of trace element contaminants through various exposure pathways was evaluated by calculating both the non-cancer and the cancer risks for children and adults. The non-cancer risk assessment was calculated with the use of Equations (5)–(9), while cancer risk assessment was computed by using Equations (10)–(13). The non-cancer risk values computed for road dust were based on the reference doses (RfD) in Table 11 and the average daily dose (ADD) values summarized in Table 12. Furthermore, the slope factors in Table 11 and the average daily doses (ADD) in Table 13 were used to assess the lifetime carcinogenic risks of Cr, Ni, As, Pb, and Co in road dust. They were calculated from the average contribution of the individual trace elements in road dust for all of the exposure pathways. The overall health risk assessment of the trace elements in this study does not consider the size of the road dust particles; thus, it uses the dust content as the inhalation amount to determine the general estimation of the health risk assessment. Therefore, the findings of this study signify the utmost likely occurrence of health risks, which also provide data that are valuable for risk cautioning, monitoring, and evading pollution.

#### 3.4.1. Non-Carcinogenic Risk Assessment

**Figure 6:**
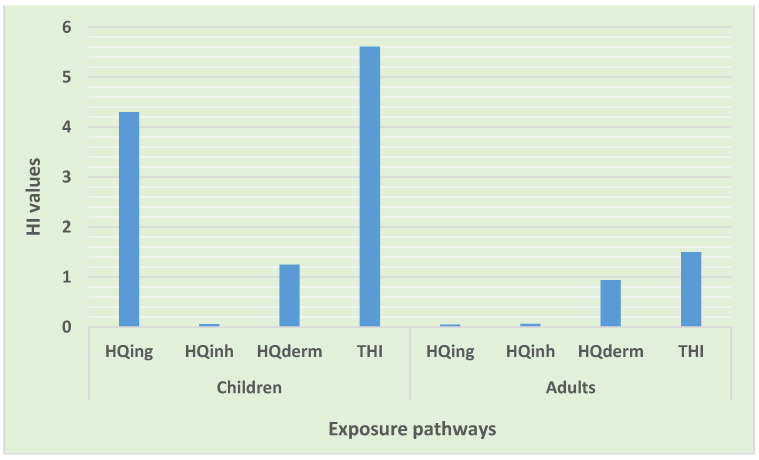
Hazard index for exposure to trace elements via various pathways.

**Figure 7:**
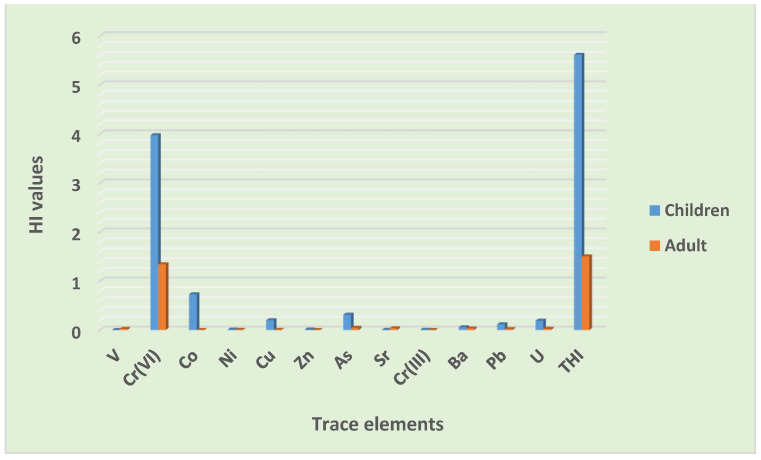
Contribution of trace elements to non-carcinogenic risk.

The total HI value in the population of children exhibited a possibility for non-carcinogenic risk (Table 12). This was driven greatly by ingestion and dermal pathways with the likelihood of non-carcinogenic risk. As shown in Figure 6, the total HI values through various exposure pathways in descending order were ingestion > dermal > inhalation, while the contribution of individual elements to the total HI was, in order, Cr(VI) > Co > As > U > V > Pb > Ba > Cu > Zn > Ni > Cr(III) > Sr (Figure 7). The discoveries of this study are similar to the findings of the studies conducted in the road dust of Viano do Castelo, Portugal [31], in Wroclaw and Katowice, Poland [53], the urbanized cities of Pakistan [9], and in Lagos metropolis, Nigeria [54].

Chromium (Cr VI) was the only element that presented a probability for non-cancer risk. Taiwo et al. [54] in Lagos metropolis, Nigeria also reported Cr as being the principal contributor to non-cancer risk in road dust, which supports the findings of this study. The outcomes of this study suggest that the children group in this informal settlement are at risk of non-carcinogenic cancer, mainly through ingestion and dermal exposure, which is a concern, considering that they have a custom of playing in the dust and sucking their fingers or hands while playing. Children may be exposed to Cr mainly through inhalation and dermal contact, as it is the only element with the possibility of non-cancer risk. Other elements and inhalation pathways showed no possibility of non-cancer risk.

In the adult population, the total HI value showed a likelihood of causing non-carcinogenic risks. All exposure pathways had no chance for causing non-carcinogenic risks, and their trends were in the order of dermal > ingestion > inhalation (Figure 6). There was also no possibility of non-cancer risk from all of the trace elements, except from Cr (VI). Their contributions to the total hazard index were as follows: Cr(VI) > As > Ba > U > V > Pb > Sr > Ni > Cu > Co > Zn > Cr(III) (Figure 7). In adults, dermal contact was the major contributor to non-cancer risk, which makes re-suspended particles a serious concern. Exposure to Cr through dermal contact may lead to the possibility of non-cancer risks among adults in this poor informal settlement. The overall level of trace elements in road dust in this informal settlement showed the possibility of non-cancer risks to the local inhabitants. Additionally, the total HI value in the children population was higher than the total HI value in the adult population, evidencing the high possibility for heavy metals to cause non-cancer risks to children compared to the adult population (Figure 6). Similar outcomes were reported by Qadeer et al. [9] in urbanized cities of Pakistan, and by Candeias et al. [31] in Viano do Castelo, Portugal.

#### 3.4.2. Carcinogenic Risk (CR) Assessment

**Figure 8:**
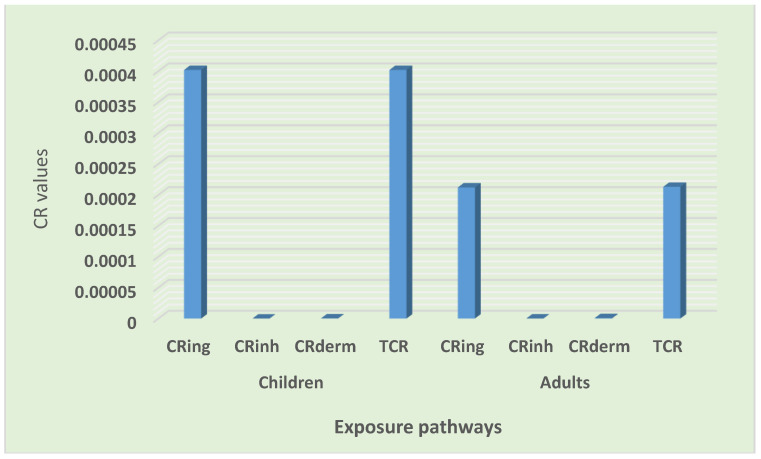
Contribution of exposure pathway carcinogenic risks.

The results of the cancer risk assessment, as summarized in Table 14, revealed that the total CR values in children and adults were between 10^−4^ and 10^−3^. Children were at a higher risk than adults. Furthermore, the lifetime cancer risk value for the entire population was also between 10^−4^ and 10^−3^. This value is considered to be a high risk, suggesting a concern for the residents regarding the possible CR of trace elements in road dust. Only the ingestion pathway exhibited the probability for cancer risk to both children and adults. The total cancer risks through various exposure pathways were as follows: ingestion > dermal > inhalation and ingestion > dermal > inhalation, for both children and adults, respectively (Figure 8).

**Figure 9:**
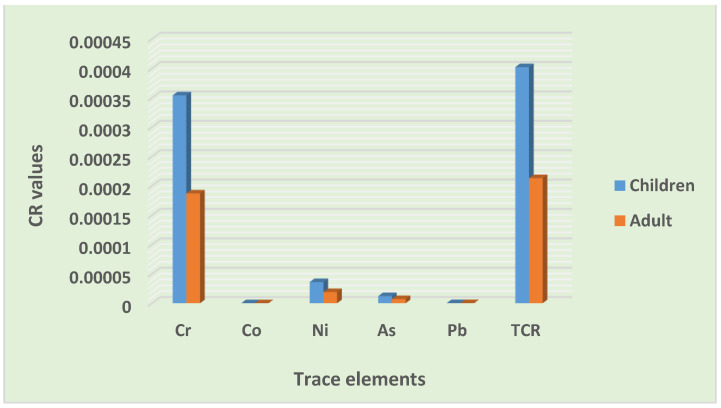
Contribution of trace elements to cancer risks.

**Figure 10:**
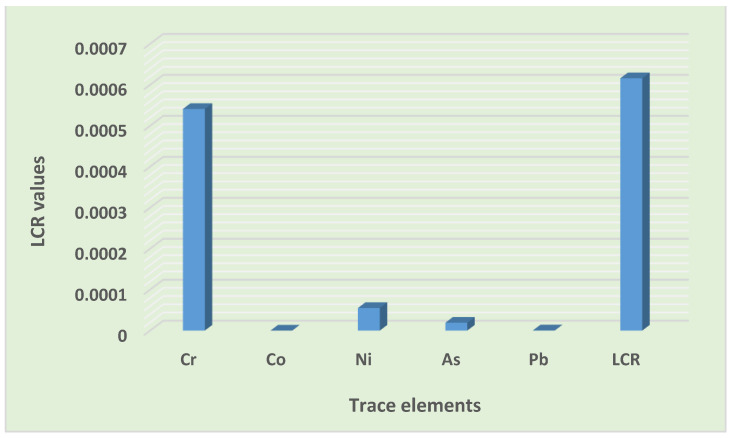
Contribution of trace elements to lifetime carcinogenic risks.

Furthermore, all of the trace element cancer risks values were within the acceptable limit in both the children and adult groups, except for the Cr risk value. The CR values of all of the elements were as follows: Cr > Ni > As > Pb > Co and Cr > Ni > As > Pb > Co for children and adults, respectively (Figure 9. According to various researchers, including Qadeer et al. [9] in the urbanized cities of Pakistan, and Candeias et al. [31] in Viano do Castelo, Portugal, the ingestion of road dust in the children group was the leading contributor to cancer risk, which is comparable to the outcomes of this study. Similar findings confirming Cr to be the main driver of carcinogenic risk were also reported by Dat et al. [51] in the street dust of a metropolitan area in Southern Vietnam. Similarly, Dat et al. [51], for the street dust of a metropolitan area in Southern Vietnam, and Taiwo et al. [54], for Lagos metropolis, Nigeria, elucidated the ingestion pathway and determined Cr as being the leading contributor to the total cancer risk among adults. From the results of the cancer risk assessment, it was clear that trace elements in road dust had the possibility for contributing to lifetime carcinogenic risks for the entire population, and Cr was the leading contributor (Figure 10). Children were at a high risk of cancer compared to the adult group (Figure 9). The community should be educated on the importance of the environment, pollution, waste management, and health. Remediation, cleaning, and the regular monitoring of heavy metals is desirable for the safety of the inhabitants and the sustainability of the settlement.

### 3.5. Health Implications Associated with Trace Elements Exposure in Road Dust

Among the examined elements, only six trace elements were above their average shale values, which is a health concern for the local population, particularly children. As reported by Jin et al. [66], outdoor play areas represent important exposure situations for children in many urban settlement areas. Hassaan et al. [57], stated that trace elements at high concentrations are toxic. The toxicity of trace elements in this study can be listed as Cr > Ba > Zn > Zr > Cu > Pb, and their exposure may lead to serious health implications to the local inhabitants. Cr may trigger liver disorder and irritation [11]. Barium (Ba) may lead to renal failure and lung sclerosis [67]. Exposure to zinc (Zn) may cause risks of prostate cancer, and exhaustion [12]. Zirconium (Zr) may lead to pulmonary effects and hoarseness [68]. Copper (Cu) is associated with dizziness and stomach cramps [12]. Furthermore, exposure to lead (Pb) may cause hormonal changes and reduced potency in males [69].

## 4. Conclusions

This study was aimed at determining the influence of urban informal settlements on trace element accumulation in road dust from Ekurhuleni Metropolitan Municipality, South Africa, and their health implications. The outcomes of the study have revealed that informal settlement activities have considerable influences on the accumulation of trace elements in road dust during the winter season. In this poor informal settlement, major elements (SiO_2_ and P_2_O_5_) and trace elements (Cr, Cu, Zn, Zr, Ba, and Pb) were particular health concerns as they were above their corresponding average shale values. In particular, Cr was a major health concern to the inhabitants, as its level of accumulation in the road dust was high. Furthermore, the level of trace elements in road dust exhibited a possibility for non-cancer risks and cancer risks to the entire population, and Cr was the major driver for both non-carcinogenic and carcinogenic risks for the entire population. Cleaning and the regular monitoring of heavy metals in poor urban informal settlements is desirable for the safety of the inhabitants and the sustainability of the settlement. The study will provide valuable scientific data on urban informal settlement geochemistry and health risks, which will be used as a reference value and for remediation measures. The overall health risk assessment of trace elements in this study did not take into account the size of the road dust particles; thus, future work should cover the variation in particle size distribution and morphology over different seasons of the year.

## Figures and Tables

**Table 1:** Location and characteristics of sampling points.

Sample ID.	Description	GPS Coordinates	
		Latitude	Longitude
RD01	At one of the major roads (Madiba drive)	25°58′22.1″ S	28°13′27.1″ E
RD02	At the southward drive, near the park	25°58′00.3″ S	28°12′57.6″ E
RD03	At the community taxi rank	25°58′51.4″ S	28°13′22.1″ E
RD04	Next to primary school gate	25°58′50.7″ S	28°13′30.1″ E
RD05	Within the community shopping centre or mall	25°58′33.0″ S	28°13′42.2″ E

**Table 4:** Statistical analysis of trace elements concentration (mg/kg) in road dust samples with average shale values (mg/kg) and South African soil screening values (mg/kg).

Elements	LOD	RD01	RD02	RD03	RD04	RD05	Min-Max	Mean	±SD	ASV	SASSV
As	1	9	10	4	4	9	4–10	7.2	2.9	13	23
Ba	17	563	636	546	729	654	546–729	625.6	73.9	580	n.a
Co	3	16	15	17	20	19	15–20	17.4	2.1	19	300
Cr	4	493	283	694	1088	629	283–1088	637.4	297	90	6.5
Cu	4	125	42	45	43	50	42–125	61	35.9	45	1100
Nb	1	9	8	9	8	9	8–9	8.6	0.5	11	n.a
Ni	4	37	32	53	80	43	32–80	49	19	68	620
Pb	2	32	17	30	41	34	17–41	30.8	8.8	20	110
Rb	2	61	82	59	72	56	56–82	66	10.8	140	n.a
Sc	3	7	8	4	5	5	4–8	5.8	1.6	13	n.a
Sr	2	156	153	99	81	112	81–156	120.2	33.2	300	n.a
Th	3	6	6	5	2.9	3	2.9–6	4.58	1.5	12	n.a
U	3	2.9	2.9	2.9	2.9	2.9	2.9–2.9	2.9	0	3.7	n.a
V	5	57	55	76	75	82	55–82	69	12.2	130	150
Y	1	15	14	14	14	15	14–15	14.4	0.5	26	n.a
Zn	2	700	131	147	67	114	67–700	231.8	263.4	95	9200
Zr	1	164	167	221	172	227	164–227	190.2	31.1	160	n.a

Notation: LOD = limit of detection; RD = road dust; n.a = not available; SD = Standard deviation; ASV = Average shale value; SASSV = South African Soil Screening Value; Min = Minimum; Max = Maximum. Average shale values [35], SASSV [36].

**Table 5:** Comparison of trace elements (mg/kg) in road dust with other global cities.

Trace Elements (mg/kg)	City and Country									
	Ekurhuleni (South Africa)	Dhaka City (Bangladesh)	Tyumen (Russia)	Viana do Castelo (Portugal)	Barbican Downtown (China)	Luanda (Angola)	Ahvaz (Iran)	Seoul (Korea)	Xian (China)	Villavicencio (Columbia)
As	7.2	-	5.7	35	11.7	5	6	24.9	-	-
Ba	625.6	-	317.1	390	748.2	351	-	570	-	-
Co	17.4	-	39.6	-	13.7	2.9	13	17.9	34.1	-
Cr	637.4	-	507.9	210	175.2	26	57	130	175.3	9.4
Cu	61	59.3	57.4	260	50.9	42	45	351	48.9	126.3
Nb	8.6	-	6.6	-	11.9	131	-	-	-	-
Ni	49	-	632.1	16	21	10	58	62	28.3	5.3
Pb	30.8	59.6	33.9	86	93.5	1.7	86	214	97.6	87.5
Rb	66	88.1	27.1	240	44.2	-	-	-	-	-
Sc	5.8	-	10.1	-	-	1.3	-	-	-	-
Sr	120.2	289.9	147.3	190	186.5	172	-	-	-	-
Th	4.6	-	1.9	-	-	1	-	-	-	-
U	2.9	-	1.1	3.6	-	-	-	-	-	-
V	69	-	66.4	15	69.3	20	184	35	55.8	-
Y	14.4	-	7.3	-	18.6	-	-	-	-	-
Zn	231.8	189	160.8	1180	272	317	999	1476	164.9	133.3
Zr	190.2	165.6	60.7	360	120.1	-	-	-	-	-
References	Current study	[45]	[46]	[31]	[33]	[27]	[47]	[48]	[42]	[49]

**Table 6:** Geo-accumulation (Igeo) values of trace element contamination in road dust.

Igeo Values of Heavy Metals in Road Samples				
Elements	Min	Max	Mean	Classification
Cr	0.63	2.42	1.42	Moderately contaminated
Zn	0.14	1.47	0.49	Uncontaminated to moderately contaminated
Pb	0.17	0.41	0.31	Uncontaminated to moderately contaminated
Cu	0.19	0.55	0.27	Uncontaminated to moderately contaminated
Zr	0.12	0.28	0.24	Uncontaminated to moderately contaminated
Ba	0.19	0.24	0.21	Uncontaminated to moderately contaminated
Co	0.16	0.21	0.2	Uncontaminated to moderately contaminated
U	0.16	0.16	0.16	Uncontaminated to moderately contaminated
Nb	0.14	0.16	0.16	Uncontaminated to moderately contaminated
Ni	0.09	0.23	0.14	Uncontaminated to moderately contaminated
Y	0.1	0.11	0.11	Uncontaminated to moderately contaminated
As	0.06	0.15	0.11	Uncontaminated to moderately contaminated
V	0.08	0.13	0.1	Uncontaminated to moderately contaminated
Sc	0.06	0.12	0.09	Uncontaminated to moderately contaminated
Rb	0.08	0.12	0.09	Uncontaminated to moderately contaminated
Sr	0.05	0.1	0.08	Uncontaminated to moderately contaminated
Th	0.05	0.09	0.08	Uncontaminated to moderately contaminated

**Table 7:** Contamination factor (CF) and pollution load index (PLI) values of trace elements in road dust.

CFs and PLI Values of Trace Elements in Road Dust Samples				
Elements	Min	Max	Mean	Classification
Cr	3.14	12.08	7.08	Very high contamination
Zn	0.7	7.37	2.44	Moderate contamination
Pb	0.85	2.05	1.54	Moderate contamination
Cu	0.93	2.78	1.35	Moderate contamination
Zr	1.02	1.42	1.2	Moderate contamination
Ba	0.94	1.26	1.08	Moderate contamination
Co	0.79	1.05	0.91	Low contamination
U	0.78	0.78	0.8	Low contamination
Nb	0.72	0.81	0.8	Low contamination
Ni	0.47	1.18	0.72	Low contamination
As	0.31	0.77	0.55	Low contamination
Y	0.54	0.58	0.55	Low contamination
V	0.42	0.63	0.53	Low contamination
Rb	0.4	0.58	0.47	Low contamination
Sc	0.31	0.61	0.45	Low contamination
Sr	0.27	0.52	0.4	Low contamination
Th	0.02	0.5	0.38	Low contamination
PLI	0.68	72.9	5.37	Very highly polluted

**Table 8:** Enrichment factor (EF) values of trace elements in road dust.

Elements	EF Average Mean Values	Enrichment Category
Cr	7.08	Significant enrichment
Zn	2.44	Moderate enrichment
Pb	1.54	Minimal enrichment
Cu	1.35	Minimal enrichment
Zr	1.2	Minimal enrichment
Ba	1.08	Minimal enrichment
Sc	1	Minimal enrichment
Co	0.92	Minimal enrichment
U	0.78	Minimal enrichment
Nb	0.78	Minimal enrichment
Ni	0.72	Minimal enrichment
Y	0.55	Minimal enrichment
As	0.55	Minimal enrichment
V	0.53	Minimal enrichment
Rb	0.5	Minimal enrichment
Sr	0.4	Minimal enrichment
Th	0.38	Minimal enrichment

**Table 9:** Correlation matrix of trace elements in road dust.

Elements	Sc	V	Cr	Co	Ni	Cu	Zn	As	Rb	Sr	Y	Zr	Nb	Ba	Pb	Th	U
Sc	1																
V	−0.91	1															
Cr	−0.72	**0.67**	1														
Co	−0.70	**0.83**	**0.89**	1													
Ni	−0.63	0.56	**0.97**	**0.81**	1												
Cu	0.37	−0.49	−0.27	−0.34	−0.37	1											
Zn	0.42	−0.57	−0.35	−0.46	−0.43	**0.99**	1										
As	**0.78**	−0.55	−0.82	−0.55	−0.86	0.36	0.37	1									
Rb	**0.63**	−0.58	−0.20	−0.32	0.01	−0.33	−0.28	0.16	1								
Sr	**0.88**	−0.85	−0.90	−0.85	−0.89	0.59	**0.65**	**0.85**	0.24	1							
Y	0.11	0.04	−0.23	0.04	−0.43	**0.67**	**0.61**	0.56	−0.63	0.38	1						
Zr	−0.75	**0.81**	0.14	0.36	0.02	−0.40	−0.42	−0.24	−0.70	−0.47	0.16	1					
Nb	−0.39	0.30	−0.15	−0.04	−0.34	0.47	0.46	0.06	−0.93	0.09	**0.67**	**0.61**	1				
Ba	−0.03	0.29	0.54	**0.67**	0.59	−0.48	−0.56	−0.12	0.43	−0.46	−0.21	−0.21	−0.70	1			
Pb	−0.66	**0.65**	**0.89**	**0.87**	**0.77**	0.11	0.00	−0.59	−0.51	−0.70	0.23	0.18	0.19	0.38	1		
Th	**0.66**	−0.86	−0.78	−0.97	−0.70	0.47	0.58	0.42	0.28	0.82	−0.05	−0.44	0.08	−0.71	−0.75	1	
U	0.00	0.00	0.00	0.00	0.00	0.00	0.00	0.00	0.00	0.00	0.00	0.00	0.00	0.00	0.00	0.00	1

Coefficients above 0.6 are in bold.

**Table 10:** Single factor analysis of variance (ANOVA) of trace element concentrations in road dust.

Analysis of Variance in Road Dust						
Source of Variation	SS	df	MS	F	*p*-Value	F Crit
Between Groups	3,245,171	16	202,823	21	5.09 × 10^−20^	1.79
Within Groups	668,540	68	9832			
Total	3,913,712	84				

**Table 11:** Reference doses for non-cancer risks and slope factors for cancer risk assessment.

Elements	RfD (mg/kg/d)			SF (mg/kg/d)			References
	RfDing	RfDinh	RfDderm	SFing	SFinh	SFderm	
As	3.00 × 10^−^^4^	3.00 × 10^−4^	1.20 × 10^−4^	1.50 × 10^0^	1.51 × 10^1^	3.66 × 10^0^	[50,62]
Ba	2.0 × 10^−^^1^	1.43 × 10^−4^	4.90 × 10^−3^	-	-	-	[27,63]
Co	3.0 × 10^−^^4^	2.00 × 10^−2^	5.40 × 10^−3^	-	8.40 × 10^−1^	-	[23,62,63,64]
Cr(VI)	3.00 × 10^−3^	2.86 × 10^−6^	6.00 × 10^−5^	5.00 × 1^−^^1^	4.10 × 10^−1^	-	[23,50,62]
Cr(III)	1.5	-	-	-	-	-	[63]
Cu	4.00 × 10^−2^	4.00 × 10^−2^	1.20 × 10^−2^	-	-	-	[23,62,65]
Nb	-	-	-	-	-	-	-
Ni	1.1 × 10^−2^	6.00 × 10^−6^	1.60 × 10^−2^	1.70 × 10^0^	9.80 × 10^0^	-	[23,62,63,65]
Pb	3.50 × 10^−3^	3.50 × 10^−3^	5.25 × 10^−4^	8.50 × 10^−3^	4.20 × 10^−2^	-	[23,62,63,65]
Rb	-	-	-	-	-	-	-
Sc	-	-	-	-	-	-	-
Sr	6.00 × 10^−1^	-	1.20 × 10^−1^	-	-	-	[27,63]
Th	-	-	-	-	-	-	-
U	2.0 × 10^−4^	-	5.10 × 10^−4^	-	-	-	[27,63]
V	5.0 × 10^−3^	7.00 × 10^−3^	7.00 × 10^−3^	-	-	-	[50,62,63]
Y	-	-	-	-	-	-	-
Zn	3.00 × 10^−1^	3.00 × 10^−1^	6.00 × 10^−2^	-	-	-	[23,63,65]
Zr	-	-	-	-	-	-	-

**Table 12:** Average daily dose (ADD), HQ, and HI values for trace elements in road dust via ingestion, inhalation, and dermal exposure pathways for children and adults.

Pathways	Average Daily Dose (ADD) for Trace Elements in Road Dust (mg/kg/day)																		Total ADD
	Sc	V	CrVI	CrIII	Co	Ni	Cu	Zn	As	Rb	Sr	Y	Zr	Nb	Ba	Pb	Th	U	
**Children**																			
**ADDing**	7.43 × 10^−5^	8.80 × 10^−4^	8.10 × 10^−3^	8.10 × 10^−3^	2.20 × 10^−4^	6.30 × 10^−4^	7.80 × 10^−4^	2.90 × 10^−3^	9.20 × 10^−5^	8.43 × 10^−4^	1.53 × 10^−3^	1.84 × 10^−4^	2.43 × 10^−3^	1.11 × 10^−4^	8.00 × 10^−3^	3.90 × 10^−4^	5.85 × 10^−^^5^	3.70 × 10^−^^5^	3.54 × 10^−^^2^
**ADDinh**	1.40 × 10^−^^9^	1.70 × 10^−8^	1.54 × 10^−7^	1.54 × 10^−7^	4.21 × 10^−9^	1.20 × 10^−8^	1.50 × 10^−8^	5.61 × 10^−8^	1.74 × 10^−9^	1.60 × 10^−8^	2.91 × 10^−8^	3.50 × 10^−9^	4.60 × 10^−8^	2.08 × 10^−9^	1.51 × 10^−7^	7.46 × 10^−9^	1.10 × 10^−^^9^	7.02 × 10^−^^10^	6.72 × 10^−^^7^
**ADDderm**	6.67 × 10^−9^	7.94 × 10^−6^	7.33 × 10^−5^	7.33 × 10^−5^	2.00 × 10^−6^	5.64 × 10^−6^	7.02 × 10^−6^	2.67 × 10^−5^	8.30 × 10^−7^	7.59 × 10^−6^	1.40 × 10^−5^	1.66 × 10^−6^	2.20 × 10^−6^	9.90 × 10^−7^	7.20 × 10^−5^	3.54 × 10^−6^	5.27 × 10^−^^7^	3.34 × 10^−^^7^	3.00 × 10^−^^4^
**ADD**	7.43 × 10^−^^5^	8.88 × 10^−4^	8.17 × 10^−3^	8.17 × 10^−3^	2.22 × 10^−4^	6.36 × 10^−4^	7.87 × 10^−4^	2.93 × 10^−3^	9.28 × 10^−5^	8.51 × 10^−4^	1.54 × 10^−3^	1.86 × 10^−4^	2.43 × 10^−3^	1.12 × 10^−4^	8.07 × 10^−3^	3.94 × 10^−^^4^	5.90 × 10^−^^5^	3.73 × 10^−^^5^	3.57 × 10^−^^2^
**Adults**																			
**ADDing**	9.94 × 10^−^^6^	1.20 × 10^−4^	1.10 × 10^−^^3^	1.10 × 10^−^^3^	2.98 × 10^−^^5^	8.40 × 10^−^^5^	1.00 × 10^−^^4^	4.00 × 10^−^^4^	1.23 × 10^−^^5^	1.13 × 10^−^^4^	2.06 × 10^−^^4^	2.47 × 10^−^^5^	3.26 × 10^−^^4^	1.47 × 10^−^^5^	1.07 × 10^−^^3^	5.28 × 10^−^^5^	7.86 × 10^−^^6^	4.97 × 10^−^^6^	4.78 × 10^−^^3^
**ADDinh**	1.50 × 10^−^^9^	1.80 × 10^−^^8^	1.66 × 10^−^^7^	1.66 × 10^−^^7^	4.52 × 10^−^^9^	1.30 × 10^−^^8^	1.60 × 10^−^^8^	6.02 × 10^−^^8^	1.90 × 10^−^^9^	1.71 × 10^−^^8^	3.12 × 10^−^^8^	3.74 × 10^−^^9^	4.94 × 10^−^^8^	2.23 × 10^−^^9^	1.62 × 10^−^^7^	8.00 × 10^−^^9^	1.20 × 10^−^^9^	7.54 × 10^−^^10^	7.23 × 10^−^^7^
**ADDderm**	4.97 × 10^−^^7^	5.92 × 10^−^^6^	5.50 × 10^−^^5^	5.50 × 10^−^^5^	1.49 × 10^−^^6^	4.20 × 10^−^^6^	5.23 × 10^−^^6^	1.99 × 10^−^^5^	6.17 × 10^−^^7^	5.66 × 10^−^^6^	1.03 × 10^−^^5^	1.23 × 10^−^^6^	1.63 × 10^−^^5^	7.40 × 10^−^^7^	5.40 × 10^−^^5^	2.64 × 10^−^^6^	3.93 × 10^−^^7^	2.49 × 10^−^^7^	2.39 × 10^−^^4^
**ADD**	1.04 × 10^−^^5^	1.26 × 10^−^^4^	1.16 × 10^−^^3^	1.16 × 10^−^^3^	3.13 × 10^−^^5^	8.82 × 10^−^^5^	1.05 × 10^−^^4^	4.20 × 10^−^^4^	1.29 × 10^−^^5^	1.19 × 10^−^^4^	2.16 × 10^−^^4^	2.59 × 10^−^^5^	3.42 × 10^−^^4^	1.54 × 10^−^^5^	1.12 × 10^−^^3^	5.54 × 10^−^^5^	8.25 × 10^−^^6^	5.22 × 10^−^^6^	5.02 × 10^−3^
**Pathways**	**Non−cancer risk values for trace elements in road dust (mg/kg/day)**																		**Total HI**
	**Children**																		
**HQing**	-	1.80 × 10^−^^1^	2.70 × 10^0^	5.40 × 10^−^^3^	7.30 × 10^−^^1^	6.00 × 10^−^^3^	1.95 × 10^−^^2^	9.67 × 10^−^^3^	3.06 × 10^−^^1^	-	2.55 × 10^−^^3^	-	-	-	4.00 × 10^−^^2^	1.11 × 10^−^^1^	-	1.90 × 10^−^^1^	4.30 × 10^0^
**HQinh**	-	2.43 × 10^−^^6^	5.38 × 10^−^^2^	-	2.10 × 10^−^^7^	2.00 × 10^−^^3^	3.75 × 10^−^^7^	1.87 × 10^−^^7^	5.80 × 10^−^^6^	-	-	-	-	-	1.05 × 10^−^^3^	2.13 × 10^−^^6^	-	-	5.69 × 10^−^^2^
**HQderm**	-	1.13 × 10^−^^3^	1.22 × 10^0^	-	3.70 × 10^−^^4^	3.52 × 10^−^^4^	5.85 × 10^−^^4^	4.45 × 10^−^^4^	6.92 × 10^−^^3^	-	-	-	-	-	1.47 × 10^−^^2^	6.74 × 10^−^^3^	-	6.55 × 10^−^^4^	1.25 × 10^0^
**HI**	-	1.81 × 10^−^^1^	3.97 × 10^0^	5.40 × 10^−^^3^	7.30 × 10^−^^1^	8.35 × 10^−^^3^	2.01 × 10^−^^2^	1.01 × 10^−^^2^	3.13 × 10^−^^1^	-	2.55 × 10^−^^3^	-	-	-	5.58 × 10^−^^2^	1.18 × 10^−^^1^	-	1.91 × 10^−^^1^	5.61 × 10^0^
**Adults**																			
**HQing**	-	2.40 × 10^−^^2^	3.67 × 10^−^^1^	7.30 × 10^−^^4^	1.49 × 10^−^^3^	8.00 × 10^−^^4^	2.50 × 10^−^^3^	1.33 × 10^−^^3^	4.10 × 10^−^^2^	-	3.43 × 10^−^^3^	-	-	-	2.00 × 10^−^^2^	1.51 × 10^−^^2^	-	2.50 × 10^−^^2^	5.02 × 10^−^^1^
**HQinh**	-	2.57 × 10^−^^6^	5.80 × 10^−^^2^	-	2.26 × 10^−^^7^	2.17 × 10^−^^3^	4.00 × 10^−^^7^	2.01 × 10^−^^7^	6.33 × 10^−^^6^	-	−	-	-	-	1.13 × 10^−^^3^	2.28 × 10^−^^6^	-	-	6.13 × 10^−^^2^
**HQderm**	-	8.46 × 10^−^^4^	9.17 × 10^−^^1^	-	2.76 × 10^−^^4^	2.62 × 10^−^^4^	4.36 × 10^−^^4^	3.32 × 10^−^^4^	5.14 × 10^−^^3^	-	8.58 × 10^−^^5^	-	-	-	1.10 × 10^−^^2^	5.03 × 10^−^^3^	-	4.88 × 10^−^^4^	9.41 × 10^−^^1^
**HI**	-	2.48 × 10^−^^2^	1.34 × 10^0^	7.30 × 10^−^^4^	1.77 × 10^−^^3^	3.23 × 10^−^^3^	2.94 × 10^−^^3^	1.66 × 10^−^^3^	4.61 × 10^−^^2^	-	3.52 × 10^−^^3^	-	-	-	3.21 × 10^−^^2^	2.01 × 10^−^^2^	-	2.55 × 10^−^^2^	1.50 × 10^0^

**Table 13:** Carcinogenic average daily dose (ADD) for trace elements in road dust via ingestion, inhalation, and dermal exposure pathways for children and adults.

Pathways	Cancer Average Daily Dose (ADD) for Trace Elements in Road Dust (mg/kg/day)																		Total ADD
	Sc	V	CrVI	CrIII	Co	Ni	Cu	Zn	As	Rb	Sr	Y	Zr	Nb	Ba	Pb	Th	U	
**Children**																			
**ADDing**	7.43 × 10^−^^5^	8.80 × 10^−^^4^	8.10 × 10^−^^3^	8.10 × 10^−^^3^	2.20 × 10^−^^4^	6.30 × 10^−^^4^	7.80 × 10^−^^4^	2.90 × 10^−^^3^	9.20 × 10^−^^5^	8.43 × 10^−^^4^	1.53 × 10^−^^3^	1.84 × 10^−^^4^	2.43 × 10^−^^3^	1.11 × 10^−^^4^	8.00 × 10^−^^3^	3.90 × 10^−^^4^	5.85 × 10^−^^5^	3.70 × 10^−^^5^	3.54 × 10^−^^2^
**ADDinh**	1.40 × 10^−^^9^	1.70 × 10^−^^8^	1.54 × 10^−^^7^	1.54 × 10^−^^7^	4.21 × 10^−^^9^	1.20 × 10^−^^8^	1.50 × 10^−^^8^	5.61 × 10^−^^8^	1.74 × 10^−^^9^	1.60 × 10^−^^8^	2.91 × 10^−^^8^	3.50 × 10^−^^9^	4.60 × 10^−^^8^	2.09 × 10^−^^9^	1.51 × 10^−^^7^	7.46 × 10^−^^9^	1.10 × 10^−^^9^	7.02 × 10^−^^10^	6.72 × 10^−^^7^
**ADDderm**	6.67 × 10^−^^9^	7.94 × 10^−^^6^	7.33 × 10^−^^5^	7.33 × 10^−^^5^	2.00 × 10^−^^6^	5.64 × 10^−^^6^	7.02 × 10^−^^6^	2.67 × 10^−^^5^	8.30 × 10^−^^7^	7.59 × 10^−^^6^	1.40 × 10^−^^5^	1.66 × 10^−^^6^	2.20 × 10^−^^6^	9.90 × 10^−^^7^	7.20 × 10^−^^5^	3.54 × 10^−^^6^	5.27 × 10^−^^7^	3.34 × 10^−^^7^	3.00 × 10^−^^4^
**Total**	7.43 × 10^−^^5^	8.88 × 10^−4^	8.17 × 10^−^^3^	8.17 × 10^−^^3^	2.22 × 10^−^^4^	6.36 × 10^−^^4^	7.87 × 10^−^^4^	2.93 × 10^−^^3^	9.28 × 10^−^^5^	8.51 × 10^−^^4^	1.54 × 10^−^^3^	1.86 × 10^−^^4^	2.43 × 10^−^^3^	1.12 × 10^−^^4^	8.07 × 10^−^^3^	3.94 × 10^−^^4^	5.90 × 10^−^^5^	3.73 × 10^−^^5^	3.57 × 10^−^^2^
**Adults**																			
**ADDing**	9.94 × 10^−^^6^	1.20 × 10^−^^4^	1.10 × 10^−^^3^	1.10 × 10^−^^3^	2.98 × 10^−^^5^	8.40 × 10^−^^5^	1.00 × 10^−^^4^	4.00 × 10^−^^4^	1.23 × 10^−^^5^	1.13 × 10^−^^4^	2.06 × 10^−^^4^	2.47 × 10^−^^5^	3.26 × 10^−^^4^	1.47 × 10^−^^5^	1.07 × 10^−^^3^	5.28 × 10^−^^5^	7.86 × 10^−^^6^	4.97 × 10^−^^6^	4.78 × 10^−^^3^
**ADDinh**	1.50 × 10^−^^9^	1.80 × 10^−^^8^	1.66 × 10^−^^7^	1.66 × 10^−^^7^	4.52 × 10^−^^9^	1.30 × 10^−^^8^	1.60 × 10^−^^8^	6.02 × 10^−^^8^	1.90 × 10^−^^9^	1.71 × 10^−^^8^	3.12 × 10^−^^8^	3.74 × 10^−^^9^	4.94 × 10^−^^8^	2.23 × 10^−^^9^	1.62 × 10^−^^7^	8.00 × 10^−^^9^	1.20 × 10^−^^9^	7.54 × 10^−^^10^	7.23 × 10^−7^
**ADDderm**	4.97 × 10^−^^7^	5.92 × 10^−^^6^	5.50 × 10^−^^5^	5.50 × 10^−^^5^	1.49 × 10^−^^6^	4.20 × 10^−^^6^	5.23 × 10^−^^6^	1.99 × 10^−^^5^	6.17 × 10^−^^7^	5.66 × 10^−^^6^	1.03 × 10^−^^5^	1.23 × 10^−^^6^	1.63 × 10^−^^5^	7.40 × 10^−^^7^	5.40 × 10^−^^5^	2.64 × 10^−^^6^	3.93 × 10^−^^7^	2.49 × 10^−^^7^	2.39 × 10^−^^4^
**Total**	1.04 × 10^−^^5^	1.26 × 10^−^^4^	1.16 × 10^−^^3^	1.16 × 10^−^^3^	3.13 × 10^−^^5^	8.82 × 10^−^^5^	1.05 × 10^−^^4^	4.20 × 10^−^^4^	1.29 × 10^−^^5^	1.19 × 10^−^^4^	2.16 × 10^−^^4^	2.59 × 10−^5^	3.42 × 10^−^^4^	1.54 × 10^−^^5^	1.12 × 10^−^^3^	5.54 × 10^−^^5^	8.25 × 10^−^^6^	5.22 × 10^−^^6^	5.02 × 10^−^^3^
**LADD**	8.47 × 10^−^^5^	1.01 × 10^−^^3^	9.33 × 10^−^^3^	9.33 × 10^−^^3^	2.53 × 10^−^^4^	7.24 × 10^−^^4^	8.92 × 10^−^^4^	3.35 × 10^−^^3^	1.06 × 10^−^^4^	9.69 × 10^−^^4^	1.76 × 10^−^^3^	2.12 × 10^−^^4^	2.77 × 10^−^^3^	1.27 × 10^−^^4^	9.20 × 10^−^^3^	4.49 × 10^−^^4^	6.73 × 10^−^^5^	4.26 × 10^−^^5^	4.07 × 10^−^^2^

**Table 14:** Cancer risk assessment for trace elements in road dust via ingestion, inhalation, and dermal exposure pathways for children and adults.

Pathways	Cancer Risk Values for Trace Elements in Road Dust (mg/kg/day)					Total CR
	Cr	Co	Ni	As	Pb	
**Children**						
**CRing**	3.54 × 10^−^^4^	-	3.60 × 10^−^^5^	1.18 × 10^−^^5^	2.86 × 10^−^^7^	4.02 × 10^−^^4^
**CRinh**	5.41 × 10^−^^9^	3.03 × 10^−^^10^	9.99 × 10^−^^9^	2.26 × 10^−^^10^	2.68 × 10^−^^11^	1.60 × 10^−^^8^
**CRderm**	-	-	-	2.59 × 10^−^^7^	-	2.59 × 10^−^^7^
**CR**	3.54 × 10^−^^4^	3.03 × 10^−^^10^	3.60 × 10^−^^5^	1.21 × 10^−^^5^	2.86 × 10^−^^7^	4.02 × 10^−^^4^
**Adults**						
**CRing**	1.87 × 10^−^^4^	-	1.90 × 10^−^^5^	6.34 × 10^−^^6^	1.54 × 10^−^^7^	2.12 × 10^−^^4^
**CRinh**	2.33 × 10^−^^8^	1.30 × 10^−^^9^	4.28 × 10^−^^8^	9.69 × 10^−^^10^	1.15 × 10^−^^10^	6.85 × 10^−^^8^
**CRderm**	-	-	-	7.76 × 10^−^^7^	-	7.76 × 10^−^^7^
**CR**	1.87 × 10^−^^4^	1.30 × 10^−^^9^	1.90 × 10^−^^5^	7.12 × 10^−^^6^	1.54 × 10^−^^7^	2.13 × 10^−^^4^
**LCR**	5.41 × 10^−^^4^	1.60 × 10^−^^9^	5.51 × 10^−^^5^	1.92 × 10^−^^5^	4.40 × 10^−^^7^	6.16 × 10^−^^4^

## Data Availability

The raw data utilized in this study are obtainable upon request from the corresponding author.
